# Protective Functions of β-Alanyl-L-Histidine and Glycyl-L-Histidyl-L-Lysine Glycoconjugates and Copper in Concert

**DOI:** 10.3390/antiox14121512

**Published:** 2025-12-17

**Authors:** Irina Naletova, Enrico Rizzarelli

**Affiliations:** 1 Institute of Crystallography, National Council of Research, CNR-IC, Via P. Gaifami, 18-95126 Catania, Italy; irinanaletova@cnr.it; 2 Department of Chemical Sciences, University of Catania, V.le A. Doria, 6-95125 Catania, Italy

**Keywords:** endogenous peptides, histidine containing peptides, hyaluronan, trehalose, glycoconjugates, antioxidant, antiaggregating, SOD1 mimics, copper signaling, trophic factors

## Abstract

Two endogenous peptides, β-alanyl-L-histidine, named carnosine (Car), and glycyl-L-histidyl-L-lysine (GHK), derived from the matricellular protein Secreted Protein Acidic and Rich in Cysteine (SPARC), share many beneficial functions. The hydrolytic enzyme carnosinase for Car and the low stability for GHK can put into question their antioxidant, antiaggregating, and anti-inflammatory properties. The glycoconjugates of Car with a di- (trehalose, Tre) or polysaccharide (hyaluronan, HA) inhibit carnosinase, while the synthesis of HAGHK derivatives increases the tripeptide stability and protects/delays the biopolymer degradation. A synergic effect between the two components of the glycoconjugates is evident in their consequently preserved protective features. TreCar, HACar, and HAGHK maintain the copper-binding ability of the peptides alone, and the saccharides potentiate the Cu,Zn-superoxide dismutase-like ability of the copper(II) complexes with the glycoconjugates. These peptide derivatives behave as copper ionophores, utilizing Cu^2+^ present in the culture medium; also, an increase in the metal intracellular level occurs with a consequent stimulation of the copper-driven signaling pathways that produce the expression/release of trophic (Brain-Derived Neurotrophic Factor, BDNF, and Bone Morphogenetic Protein 2, BMP-2) and angiogenic (Vascular Endothelial Growth Factor, VEGF) proteins. Copper chaperons for SOD1, CCS, and Antioxidant 1 (Atox-1) are the copper chaperones that act as transcription factors.

## 1. Introduction

Cellular homeostasis needs redox homeostasis that requires a balance between the production of reactive oxygen species (ROS) and the endogenous antioxidant system [1], with ROS being also involved in physiologic signal transduction and cell protection [2].

Mitochondrial and non-mitochondrial ROS-generating enzymes, including nicotinamide adenine dinucleotide phosphate (NADPH) oxidase (Nox), xanthine oxidase (XO), cytochrome P450 from endoplasmic reticulum (ER), and flavin oxidases from peroxisomes, are responsible for endogenous production of ROS [3,4]. The mitochondrial respiratory chain and Nox systems are the main players.

**Scheme 1:**
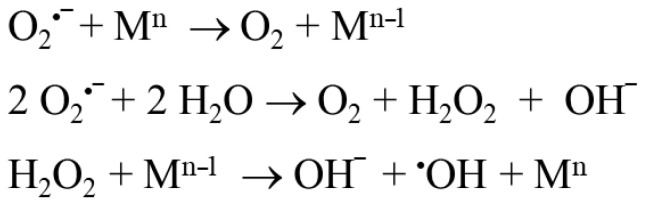
Fenton and Haber–Weiss reaction.

**Figure 1:**
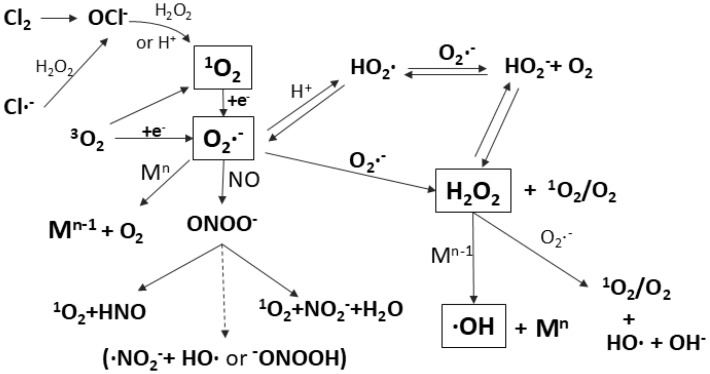
Main reactions for generating, consuming, and interconverting ROS.

Two types of ROS are produced, which include both radical species, such as superoxide anion (O_2_ ·−) and hydroxyl radical (·OH), and non-radical species, such as hydrogen peroxide (H_2_O_2_). High-reactive molecules such as hydroxyl radicals and peroxynitrites are generated when H_2_O_2_ undergoes Fenton and Haber–Weiss reactions (Scheme 1) [5]. When H_2_O_2_ and Fe(II)/Cu(I) are the only reactants, the first reaction is just the first step of a complex process. (Figure 1)

Superoxide (O_2_^−^.) is generated from O_2_ as a by-product of the respiratory chain complex from the mitochondrial electron transport chain (ETC) or by NADPH oxidase. The superoxide radical is then dismutated into H_2_O_2_ by the enzyme superoxide dismutases (SODs), including cytosolic Cu,Zn-SOD (SOD1) and mitochondrial Mn-SOD (SOD2) [6] and the extracellular isoform, SOD3 [7]. Moreover, catalase (CAT), glutathione peroxidase (GPx), and peroxiredoxins (PRX) [8] detoxify hydrogen peroxide. In addition to enzymes committed to ensuring redox homeostasis [9,10], cells also have an extensive array of non-enzymatic antioxidants, which act as an additional layer of defense. Some examples include glutathione, vitamin C (ascorbic acid), and vitamin E (α-tocopherol) [11].

ROS directly interacts with critical signaling molecules, stimulating diverse cellular processes such as proliferation and survival (mitogen-activated protein kinases, MAP kinases [12,13]; phosphoinositide 3-kinase, PI3 kinase [14,15]; PTEN [16,17]; and protein tyrosine phosphatases [18]), ROS homeostasis and antioxidant gene regulation (thioredoxin [19]; peroxiredoxin [20]; Redox factor-1, Ref-1 [21]; and nuclear factor erythroid 2-related factor 2, Nrf-2 [22,23]), and mitochondrial oxidative stress, apoptosis, and aging (the Shc adaptor protein family component, p66Shc [24]; and Ataxia–telangiectasia mutated, ATM [25]).

However, the unbalance of redox homeostasis leads to oxidative damage and cellular dys-homeostasis, associated with various pathological processes, including: (i) neurodegenerative pathologies [26], by accelerating neuron function alteration and neuronal apoptosis [27]; (ii) cardiovascular diseases progression by favoring endothelial illness [28]; (iii) cancer evolution by activating different signaling pathways [29,30]; and (iv) immune system worsening [31].

The brain with its numerous and incurable diseases (Alzheimer’s diseases, Parkinson’s disease, amyotrophic lateral sclerosis pathology, etc.) is highly vulnerable to oxidative stress due to: (i) high oxygen consumption; (ii) significant levels of redox-active metals, such as iron or copper, which can catalyze ROS formation; (iii) high levels of polyunsaturated fatty acids in its cell membranes, which can act as substrates for lipid peroxidation [32]; and, (iv) relatively low levels of GSH, which can play a limited role as an endogenous antioxidant to scavenge ROS [33].

In recent years, advances in redox biology have revealed potential therapeutic targets and strategies for modulating oxidative stress (OS) [4], such as the use of antioxidants, ROS scavengers, and signaling pathway inhibitors, some of which are undergoing clinical evaluation [34,35,36].

Current antioxidants applied in clinics for therapeutic applications are mainly molecular antioxidants, including N-acetylcysteine, edaravone, coenzyme Q10, vitamin C, etc. [37,38,39,40,41]. However, from a perspective of chemical reaction, these molecular antioxidants can only act as reactants to interact with ROS; thus, after the redox reaction, these antioxidants are inactivated, leading to a non-sustainable antioxidative effect, largely compromising therapeutic outcome [42].

Among the increasing number of peptide-based potential drug therapies that were proposed, peptides comprising 2–4 amino acid residues are defined as ultrashort peptides [43]. β-alanyl-L-histidine, named carnosine (Car), and glycyl-l-histidyl-l-lysine (GHK) are endogenous peptides that belong to this class of peptides, holding several important beneficial properties, including antioxidant, antiglycating, anti-inflammatory, and neuroprotective capacities [44,45,46,47].

## 2. Endogenous Histidine-Containing Peptides

### 2.1. β-Alanyl-L-Histidine, Carnosine

**Figure 2:**
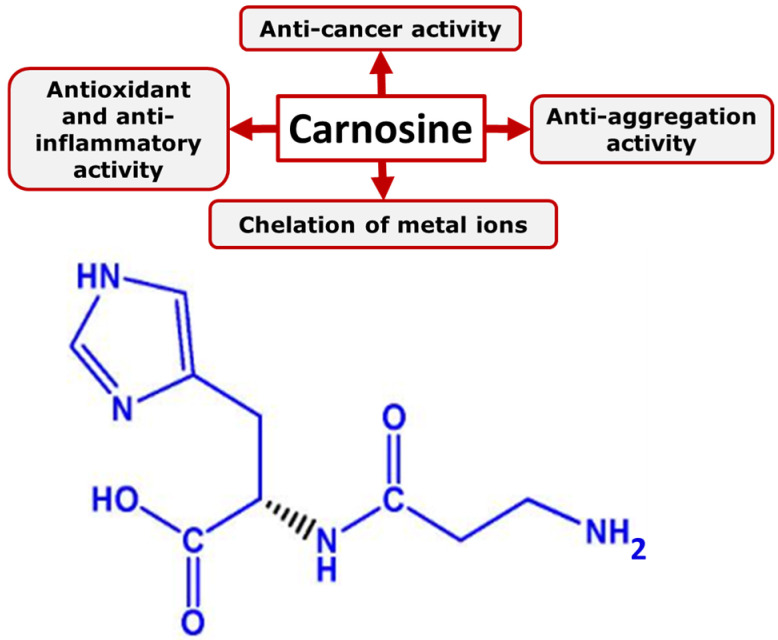
Chemical structure of carnosine.

Carnosine (β-alanyl-L-histidine, Car) (Figure 2) represents the archetype of a larger family of other subsequently isolated carnosine-related dipeptides [48], as anserine (β-alanyl-3-methyl-histidine, Ans) and homocarnosine (γ-aminobutyryl-L-histidine, HCar), which are the three most representative histidine dipeptides [49,50,51], widely present in mammal tissues [52].

The dipeptide carnosine is synthesized from its components, histidine and β-alanine, by carnosine synthetase (EC 6.3.2.11) [53]. This primarily cytosolic enzyme, present in the brain and muscles, is an ATP-dependent enzyme capable of also synthesizing homocarnosine and anserine [54]. Car uptake is an energy-assisted process regulated by means of the PepT2 transporter [55], present in the lung, the spleen, and, to a greater extent, the kidneys [56]. Unlike other endogenous peptides, Car does not undergo the hydrolytic attack of many common peptidases, but can be degraded by two human isoforms of metalloproteases, named carnosinases [57]: the serum-circulating form CN1 (EC 3.4.13.20) [58] and the cytosolic isoform CN2 (EC 3.4.13.18) [59,60]. The CN1, mainly expressed in the central nervous system, is significantly selective and hydrolyses mainly carnosine, with homocarnosine to a lesser extent [61], and its activity increases with age [58]. CN2, present in different human tissues and in the rodent brain, is a non-selective dipeptidase and hydrolyses a large number of dipeptides, but not homocarnosine [61]. The active site of CN2 contains two metal ions and requires at least an Mn^2+^ equivalent for hydrolytic activity, whereas CN1 commonly utilizes Zn^2+^ and is activated by Cd^2+^.

Carnosine is present at micro-to-millimolar concentrations in different tissues (heart, kidney, gastrointestinal tissues, etc.); high concentrations are found in skeletal muscle and in the olfactory bulb, where their concentrations are in the mM range [62], while blood and the cerebrospinal fluid contain a low amount of Car (below 100 nM) [63].

Car is well known for its intracellular proton-buffering ability [64,65] and antioxidant features [66], including: carbonyl group sequestering [67], metal binding [68], superoxide scavenging [69], and cytotoxic α,β-unsaturated aldehyde quenching [70]. Furthermore, Car shows protective effects during nitrosative stress [71], forming carnosine/NO and carnosine/NO_2_ adducts [72]. The dipeptide also inhibits the aggregation and fibril formation of different proteins, altering the non-covalent bonds between the peptide domains, which drive the oligomerization processes [73,74,75,76]. In addition, carnosine displays anti-glycating activity, avoiding the formation of advanced glycation end products (AGEs) [77,78] and DNA-protein cross-links [79]. Carnosine also protects ceruloplasmin’s antioxidant activities [80] and SOD1 [81]. Carnosine complex with copper (II) ion acts as a SOD1 mimic; this dipeptide capacity is of biological relevance as its high concentration in the skeletal muscle (mM) impacts the copper amount, which represents one-third of the total copper in the body (20–47 mmol/kg) [82].

Different studies report on the anti-inflammatory and immunomodulatory effects mediated by Car [44,83,84]. Dipeptide shows a protective effect from inflammation by decreasing ROS, nitric oxide, and inflammatory cytokine production, and increasing glutathione peroxidase-mediated enzymatic reactions. Car relieves interleukin1α-induced dry-eye disease in rabbits [85] and decreases renal interleukin-6 (IL-6) and the tumor necrosis factor-alpha (TNF-α) levels in rats during nickel-induced nephrotoxicity [86]. In addition, the complex of zinc(II) with carnosine blocks lipopolysaccharide-driven inflammatory responses by repressing nuclear factor kB (NF-κB) activation [87] or inducing the nuclear factor erythroid 2-related factor 2(Nrf2)-heme oxygenase (HO-1) signaling pathway [88] in RAW 264.7 murine macrophages. Zn-carnosine and some of its derivatives protect gastrointestinal districts by inhibiting NF-κB signaling existing in the colon, reducing interleukin-8 release [89,90], inducing antiulcer effects and wound healing activity [91,92], and preventing inflammatory cell infiltration due to induced colitis in rats [93].

Oxidative stress is widely recognized to be involved in the etiology and pathogenesis of major human diseases [94], including neurodegenerative diseases [95], cardiovascular diseases [96], and diabetes [97]; thus, Car with its well-known direct and indirect antioxidant activity has been assayed in cellular lines and in small animals as a potential therapeutic agent against many degenerative disorders [47,98,99,100,101,102,103,104,105,106,107,108,109,110,111,112,113,114,115,116,117].

The protective effects reported in the above-cited studies testify to the pleiotropic ability of the natural dipeptide and its analogs, questioning the attribution of these effects to the antioxidant features alone, and highlighting their potential therapeutic abilities against diverse disorders [118], thus justifying the designation as enigmatic peptides [119].

Car shows metal-binding features and its complexes with copper(II) and zinc(II) ions [120] provide protective effects in vitro and in vivo [82,121,122,123,124]. The attribution of these capacities to the dipeptide’s ability to sequester metal ions as Cu^2+^ [120] usually overlooks Car’s ionophore ability [125].

Small molecules that can both form metal complexes by binding d-block metal ions and favor their cellular uptake are named ionophores [126]. This class of metal ligands [127,128] perturbs the cell metallome [129], inducing a redistribution of metal ions among various intracellular districts and facilitating their uptake where they are required [130]. Furthermore, ionophores can alter the distribution of a bio-metal among the static metallome (metal ions strongly bound to proteins) [131] and dynamic metallome (metal ions present as labile and exchangeable species) [132]. The dynamic metallome is structured in metal-chaperones [133], metal-transporters [134], transcription factors (TFs) [135], storage molecules, such as metallothioneins (MTs) [136], and low molecular weight molecules like glutathione [137] which altogether guarantee the metallostasis (metal homeostasis) and the redox homeostasis of the cells [138]. This complex network is better known for zinc and copper, which are reduced to Cu^+^ before their cellular uptake. Copper transporter 1 (Ctr1), SLC 31A1, of the family of solute carriers [139] is the main membrane protein that selectively transports Cu^+^ into the cell [140] and regulates the intracellular level of the metal ion within the homeostatic range. Different cytosolic chaperones transfer Cu^+^ to specific partners: (i) the superoxide dismutase copper chaperone (CCS) to SOD1; (ii) cytochrome c oxidase 17 (COX17) to SCO1 and SCO2, which mediate copper incorporation into cytochrome c oxidase [141]; and (iii) antioxidant 1 (Atox-1) that delivers copper to two P-type ATPases, ATP7A and ATP7B [142,143].

Recently, it was shown that cellular incubation of Car [144] and some small peptides [145,146] induces an increase in cytosolic copper levels, acting as ionophores. These ligands utilize the metal ion present in the cell culture medium and the serum used as its supplement, whose total amount ranges between 10^−2^ μM and μM [147]. This Car behavior influences mainly the expression of two related components of metallostasis: Ctr1 [148] and the Cu-responsive transcription factor Sp1 (specificity protein 1) [135], which regulate the copper homeostasis of cells. Noteworthy, Car also passes through the blood–brain barrier and produces the secretion of neurotrophins (NTs) [149] in glial cells [150], stimulating different cerebral activities and decreasing cognitive decline along the brain–gut axis, by activation of the transcription factor, cAMP response element-binding protein (CREB) [151]. This transcription factor, in turn, provokes the synthesis and the release of the NT, Brain-Derived Neurotrophic Factor (BDNF) [152], also from intestinal epithelial cells [153,154]. BDNF plays a key role in normal brain development [155] and affects synaptic plasticity, learning and memory, neurogenesis, and axonal regeneration [156,157,158]. The binding of BDNF to its tyrosine kinase receptor (TrkB) induces its dimerization and autophosphorylation [159]; the phosphorylated TrkB activates different kinase cascade pathways: phosphatidylinositol 3-kinase, mitogen-activated protein kinase, and phospholipase C-c [160,161,162]. All these pathways converge on CREB, a major mediator of neuronal NT activities [163] as also validated by the behavior of the linear and cyclic peptide fragments of different NT N-terminus peptide domains [164,165,166,167,168,169], which imitate the signaling effect of NTs, including the stimulation of tyrosine kinase cascade [170,171], in a copper- and zinc-assisted mode [172,173,174]. Noteworthy, the NT peptide fragments do not just mimic the NT mode of action [166]. The reported ionophore ability of these peptides reinforces the function, recently attributed to copper, of an intracellular signaling modulator [175,176,177,178]. The dual Car ability to act as a copper ionophore and to induce NT expression and release by means of CREB mirrors that observed with NT peptide fragments. However, it is well known that the potential therapeutic action of Car is drastically hampered by its hydrolysis due to serum [57,61] and tissue [59] carnosinase enzymes [179].

Recent reviews report on innovative Car delivery systems, including vesicular systems and hybrid nanoparticles [116], carnosine derivatives and conjugates with small and polymeric carbohydrates [113,114], as well as carnosinase inhibitors [180], which represent new approaches to overcome the limitations of the peptide’s pharmacological use.

Car and some of its analogs (homocarnosine and anserine) conjugated with mono-, di (trehalose), cyclic (β-cyclodextrin), and linear poly-saccharides (hyaluronic acid) show that the glycoconjugation protects the dipeptide from carnosinase hydrolysis, thus improving the availability of carnosine [181,182,183,184]. Here, we demonstrate the abilities of some Car glycoconjugates [113,184,185,186] to display antioxidant, antiaggregating, and neuroprotective functions, which were potentiated by the saccharides (trehalose or hyaluronic acid). The glycoconjugates maintain the metal-binding capacity of the peptide, favoring copper signaling, responsible for the expression of neurotrophic factors such as BDNF and Vascular Endothelial Growth Factor (VEGF) [187,188,189,190]. These trophic features were also validated in carnosine-biofunctionalized hydroxyapatite [191].

### 2.2. Glycyl-L-Histidyl-L-Lysine, GHK

**Figure 3:**
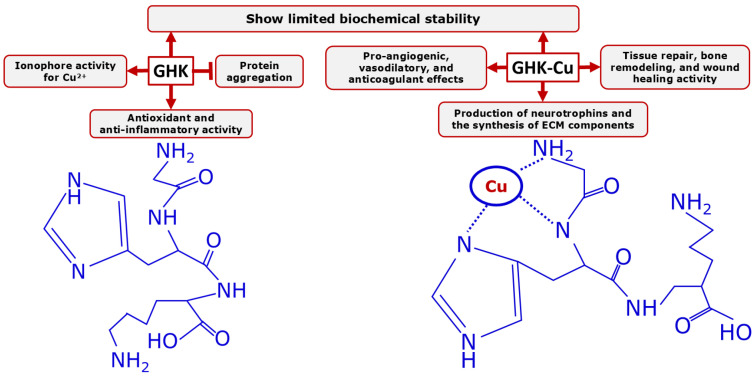
Chemical structure of glycyl-L-histidyl-L-lysine (GHK) and its copper (II) complex (GHK-Cu).

Glycyl-L-histidyl-L-lysine (GHK), structurally related to carnosine, is a natural tripeptide (Figure 3) first isolated from human plasma and also found in saliva and urine [192]. It is produced by the degradation of SPARC, a protein associated with the extracellular matrix (ECM), during the ECM degradation to aid in tissue remodeling by enhancing angiogenesis levels [193]. It shows high affinity for copper [194], forms a copper complex, GHK-Cu, and may act as a metal transporter for cellular uptake [195,196].

GHK shows a protective function quenching α,β-4-hydroxy-trans-2-nonenal [197] and acrolein [198], toxic products of lipid peroxidation involved in some age-related disorders, including AD.

GHK-Cu acts as a growth-affecting factor and a wound-healing agent, inducing dermal fibroblast growth, collagen synthesis, and glycosaminoglycan production [199,200,201,202]. Furthermore, GHK/GHK-Cu enhances the expression of different trophic factors, including the Neurotrophic Growth Factor (NGF) and Neurotrophins (NT-3 and NT-4) in the regenerating tissues [203]. Furthermore, Mesenchymal Stem Cells (MSC) treated with the tripeptide show a dose-dependent enhancement of VEGF levels [204], and angiogenesis is induced in rabbit models by copper tripeptide acting as a chemoattractant for capillary cells [205]. In addition, GHK-Cu at a low concentration (1 nM) enhances the expression of basic fibroblast growth factor (bFGF) [206] and VEGF in irradiated human dermal fibroblasts, supporting vessel formation and blood flow into damaged tissues [207]. Different studies report on the roles of GHK/GHK-Cu(II) in tissue repair, bone remodeling, and wound healing [208,209,210]. Moreover, GHK at concentrations of 10 µM or less behaves as an endogenous antioxidant, decreasing the tert-butyl hydroperoxide-induced ROS levels in Caco-2 cells [211], while GHK-Cu exerts antioxidant and anti-inflammatory functions, attenuating cigarette smoke-induced emphysema by downregulating NF-κB inflammation activity and upregulating the antioxidant Nrf2/Keap1 in lung tissues [212]. In a LPS-induced (LPS = lipopolysaccharide) inflammation model in vivo and in vitro, GHK-Cu loaded into hydroxyapatite microspheres (HAPs) and mixed with carboxymethyl cellulose, glycerol, and water forms a gel able to decrease inflammatory agents, ROS levels, and increase SOD activity [46]. The copper(II) complex with GHK shows protective effects in bleomycin-induced pulmonary fibrosis, significantly decreasing the levels of proinflammatory cytokines and oxidative stress, by blocking the progression of epithelial–mesenchymal transition and repressing Transforming Growth Factor-beta1 (TGFβ1) and Suppressor of Mothers against Decapentaplegic2/3 (Smad2/3) signaling pathways [213]. The anti-aging effect of GHK/GHK-Cu was initially restricted to aged-skin remodeling [209] and regeneration [205] until Pickart et al. highlighted that GHK can activate numerous genes involved in nervous system physiology, development, and maintenance [214], using the Broad Institute’s Connectivity Map (cMap) [215]. Specifically, this study reported that the expression of five genes was induced, while no genes were suppressed by GHK. Moreover, the tripeptide behaves as a gene regulator inhibiting histone deacetylase proteins (HDACs) and recalling the features of HDAC inhibitors, which function as neuroprotective and neurodegenerative agents in animal models of brain diseases [216]. In this context, it should be further stressed that though data are collected for GHK without copper, the possibility remains that the actual gene regulator is GHK-Cu formed in the culture media, due to the high affinity of GHK for Cu^2+^, which can allow obtaining copper from other biological molecules present in the media. These preliminary findings are supported by a more complete study that reports the effect of GHK on gene expression important for nervous system functioning and cognitive decline, as well as reviews genetic and laboratory data relevant to different biological processes and changes in gene expression for different cells [217]. Moreover, GHK can reverse memory impairment in aging mice by targeting anti-inflammatory, antioxidant, and epigenetic pathways [218], thus becoming a good candidate as a therapeutic agent in the prevention and treatment of age-associated neurodegeneration and cognitive capacity decrease. This potential effect of GHK-Cu is put in evidence in transgenic 5xFAD mice, a mouse genotype characterized by the presence of amyloid plaques [219]. Transgenic 5xFAD mice are characterized by amyloid neuropathology, similar to the amyloid-plaque pathogenesis observed in AD patients [220], and associated with synaptic alteration and relevant cognitive decline [221]. Intranasal treatment of GHK-Cu [45] provokes a reduction in amyloid plaques and a decrease in monocyte chemoattractant protein-1 (MCP-1), a key mediator in AD inflammatory processes [222]. These findings indicate that GHK-Cu has the potential to weaken AD features, including cognitive decline, amyloid plaque accumulation, and neuroinflammation.

However, GHK is characterized by a low survival capacity in human serum, making its delivery to target sites difficult, while GHK-Cu undergoes a rapid degradation in a weakly acidic environment or peptidase attack [223,224]. To maintain the molecular integrity of the tripeptide and its complex with copper(II) ion, different carriers [46,225,226] or nanostructures are employed [227]. Moreover, GHK bioavailability can be obtained by its conjugation with chosen polymers, employing either the tripeptide as such [228,229,230] or some polypeptides encompassing the GHK motif in their sequence [231]. For this aim, hyaluronic acid (HA) [232] is an attractive biopolymer as a delivery system, but it shows scarce stability due to ROS and degradative enzymatic activity that prevent its long-term persistence [233,234]. HA-based conjugates, including small and macromolecule conjugates, cross-linked systems, cell-based HA scaffolds, HA-functionalized inorganic systems, and HA bio-coating molecules, can attenuate the biochemical instability of the biopolymer [235].

Inspired by previous reported findings that: (a) HA and GHK/GHK-Cu, each by itself, show beneficial properties, including antioxidant, antiaggregating, anti-inflammatory, and neuroprotective functions, (b) HA can be degraded by ROS action mainly, while GHK exhibits interesting ROS scavenger properties, and (c) GHK shows limited biochemical stability when free, but it is more stable when somehow anchored to a polymer matrix, the synthesis of HAGHK conjugates was performed [146].

The bioconjugate consists of GHK and hyaluronic acid (MW 200 kDa), linked together through an amide bond between carboxyl residues spanning the polysaccharide chain and the ε-amino group of the lysine residue of the tripeptide (Figure 3). Here, the review reports on the abilities of three different HAGHK conjugates, each with a loading percentage in GHK of 5, 10, or 15% (the term “loading percentage” refers to the percentage of amidated carboxyl groups relative to the number of total carboxylates present in one HA molecule) to display potentiated the protective activities of the single components [146]. HAGHK forms copper(II) complexes, which show SOD1-like activity and induce the expression of trophic proteins VEGF and BDNF. HAGHK behaves as a copper ionophore affecting the level and the localization of both Ctr1 and Atox-1, involved in copper homeostasis and in the metal ion signaling pathways [175].

## 3. Non-Innocent Partners of β-Alanyl-L-Histidine and Glycyl-L-Histidyl-L-Lysine Glycoconjugates

### 3.1. Trehalose

**Figure 4:**
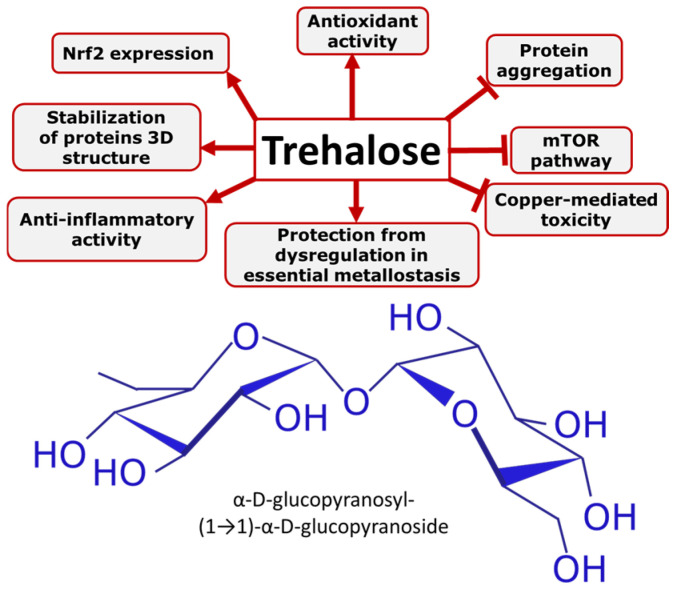
Chemical structure of trehalose.

Trehalose (Tre) is a non-reducing disaccharide consisting of two glucose units in an α,α-1,1-glycosidic linkage (Figure 4), synthesized in numerous organisms from plants and bacteria to invertebrates and yeast, except humans [236]. This disaccharide is well known to stabilize organisms, inducing anhydrobiosis and cryobiosis tolerance by protecting proteins and cell membranes against environmental stresses [236].

Moreover, Tre is considered safe for human consumption, with approval as a food ingredient in several countries [237,238].

Tre acts not only as a protector against stress but also as an effective carrier system in pharmaceuticals and biotechnology [239], ensuring stability, bioavailability, and controlled release of therapeutic molecules [240]. The role of Tre as a carrier is not only to stabilize the proteins, but also to actively contribute to maintaining biological activity and increasing the therapeutic efficacy of drugs.

Tre exhibits a broad spectrum of biological activities: antioxidant, cytoprotection, autophagy induction, modulation of metal homeostasis, anti-glycation, and anti-aging [241]. The disaccharide shows antiaggregating and anti-inflammatory properties in both cell culture and animal models [242,243] suggesting a neuroprotective role against neurodegenerative diseases [244]. The ability of Tre to interfere with amyloid beta (Aβ) fibril production and reduce its cytotoxicity indicates its beneficial role in Alzheimer’s disease (AD) [245]. The disaccharide inhibits both polyglutamine-mediated protein aggregation and alpha-synuclein fibrillation, displaying protective activities also in Huntington (HD) and Parkinson (PD) diseases [246,247].

Different reports highlight the antioxidant effects of Tre in vitro and in vivo assays [248]. Treatment with Tre in preclinical studies reveals that this antioxidant molecule significantly decreases the ROS and H_2_O_2_ levels in a dose-dependent manner [249] and upregulates gene expression of SOD, GSH, and CAT via promotion of nuclear translocation of Nrf2 [250]. Tre can interact with lipid membranes, helping preserve their integrity against lipid peroxidation, which is a major consequence of oxidative stress, leading to loss of membrane fluidity and cell death. Tre can form hydrogen bonds with phospholipids, thereby shielding membranes from radical attack [251].

In addition to cell stability and stress response, Tre shows anti-inflammatory features by suppressing the production of pro-inflammatory cytokines such as Interleukin-1β (IL-1β) and TNF-α [252] and blocking NF-κB signaling pathways [253]. By decreasing the excessive inflammatory status that characterizes the neurodegenerative disorders, the disaccharide contributes to mitigating neuroinflammation and its negative effects on neuron health [254].

Different studies report on Tre’s ability to induce autophagy [255,256], improving cellular homeostasis by the mammalian target of rapamycin complex1 pathway involvement and the transcription factor EB4 activation [257,258,259].

This capacity to degrade and recycle damaged proteins makes it a good candidate as a therapeutic agent in neurodegenerative diseases (AD, PD, and HD), where autophagy is compromised [260,261,262]. Furthermore, Tre improves both motor and cognitive outcomes in traumatic brain injury-affected mice and shows significant markers of synapse integrity and neurogenesis, such as synaptophysin, doublecortin, and BDNF, in specific brain regions [263]. Essential metal homeostasis in the brain [264,265] is strongly altered by inflammatory insults, oxidative stress, and neuronal damage, which can contribute to neurodegenerative diseases [266,267]. Recent findings indicate that the disaccharide can regulate the levels of zinc within the brain following traumatic brain injury [268]. The exact molecular mechanisms of this effect on metal ion homeostasis are not fully understood, but Tre probably moderates the depletion of copper, zinc, and iron or promotes their recovery [269]. According to current hypotheses, trehalose interacts with regulatory proteins involved in the binding, storage, chelation, and transport of metals in nerve cells. By influencing these pathways, Tre may help maintain the metal ion balance, thereby supporting a neurochemical environment conducive to neuron survival and cognitive function [270].

### 3.2. Hyaluronic Acid

**Figure 5:**
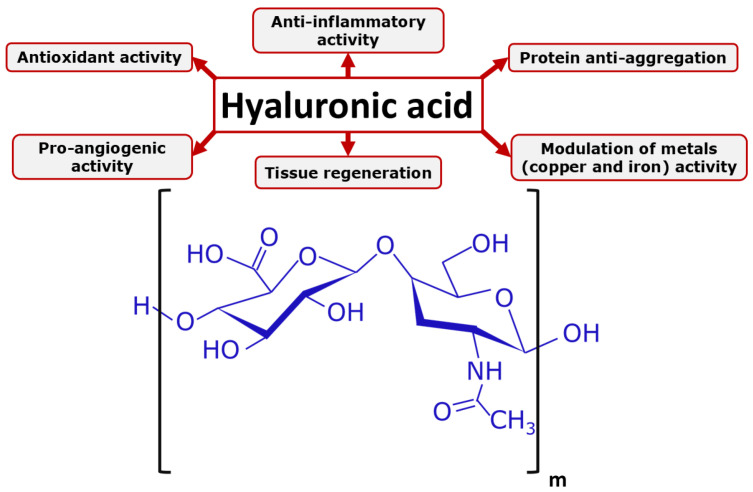
Chemical structure of hyaluronic acid, m—number of repeat units.

Hyaluronic acid (Hyaluronan) [271] is a linear polysaccharide and non-sulfated glycosaminoglycan present in the extracellular matrix, connective tissues, and body fluids. HA biopolymer (Figure 5) is formed by β-1,4- D-glucuronic acid and β-1,3-N-acetyl-D-glucosamine, which are linked β-1,3 and β-1,4 glycosidic bonds in alternative mode [272].

HA promotes tissue hydration, joint lubrication, and tissue turgor maintenance; its anionic nature, due to the carboxylic groups and the high number of hydroxyl groups, is responsible for capturing a large amount of water molecules, which explains its viscoelastic and hydrating effects [273,274]. HA is an important component of synovial fluid, where it reduces friction between the surfaces of the articular cartilage and provides resistance to compressive forces [275]. HA is synthesized by hyaluronan synthases represented by three isoforms in humans (HAS1, HAS2, and HAS3) [276,277,278], which give rise to HA chains of different sizes, low-molecular-weight (LMW) HA (6–200 kDa), medium-molecular-weight (MMW) HA (0.2–1.0 MDa), and high-molecular-weight (HMW) HA (>1 MDa) displaying differential biological activities [279,280]. HMW HA (1 MDa) is antiangiogenic, anti-inflammatory, and immunosuppressive [281] and facilitates cellular adhesion and expression of collagen, contributing to wound healing [282,283]. LMW HA induces inflammation, stimulating Toll-like receptor expression and pro-angiogenic processes, favoring cell growth and motility [284,285]. Despite its pro-inflammatory features, LMW HA shows antioxidant effects against ROS and inhibits lipid peroxidation [286]. HA shows a high cellular and tissue turnover rate caused by the degrading enzymes hyaluronidases (HYALs), with HYAL1 and HYAL2 being the main components of this family [287,288]. HYAL2 is stabilized at the cell surface by glycosylphosphatidylinositol and cleaves high molecular weight HA (HMW HA) to smaller polymeric fragments that bind to the glycoprotein CD44 [289], the main components of the HA receptor family, named hyaladherins [290]. Upon the internalization and transport of the fragments to lysosomes, further hydrolytic steps are carried out by HYAL1, which hydrolyzes the β1→4 glycosidic bond of HA, giving rise to various oligosaccharides of different lengths, the shortest of which are tetrasaccharides [287]. In addition to the hydrolysis effects of HYALs, ROS (hydroxyl radicals, peroxynitrite, and hypochlorite anion) degrade HA by randomly cleaving side groups from HA chains, causing the fragmentation of a number of small HA oligosaccharides, including monosaccharides [291,292]. In addition, ROS may both block HA biosynthesis and generate the depolymerization of the already biosynthesized HA polymers [293,294]. Due to their depolymerization effects, ROS also promote subsequent enzymatic cleavage, leading to further HA degradation [295]. Moreover, the interactions of HA with ECM molecules and cell surface receptors give rise to the signaling effects [296,297] that are mainly regulated by hyaladherins [298,299,300]. Endogenous and exogenous HA, mainly HMW HA, show antioxidant activity reducing ROS amounts in diverse cell types, including epithelial corneal cells [301], hepatic cells [302], immune cells [303], chondrocytes [304], and keratinocytes [305]. HA’s protective effects, including wound healing, tissue regeneration, anti-proliferative, anti-diabetic, anti-aging, skin repair, and cosmetic features [306,307,308], together with its properties as a delivery system [309,310], attract great interest for biomedical applications. However, HA, with its low stability and high turnover rate, presents significant restraints for therapeutic use. To overcome these issues, small antioxidant molecules [311], including carnosine [312], in a mixture with HA are required to maintain its molecular integrity and block its degradation. Bioconjugates of HA with protective moieties are able to improve HA stability and obtain derivatives with better beneficial properties [265,313,314].

## 4. Biological Features of Glycoconjugates

### 4.1. Glycoconjugates with Trehalose

**Figure 6:**
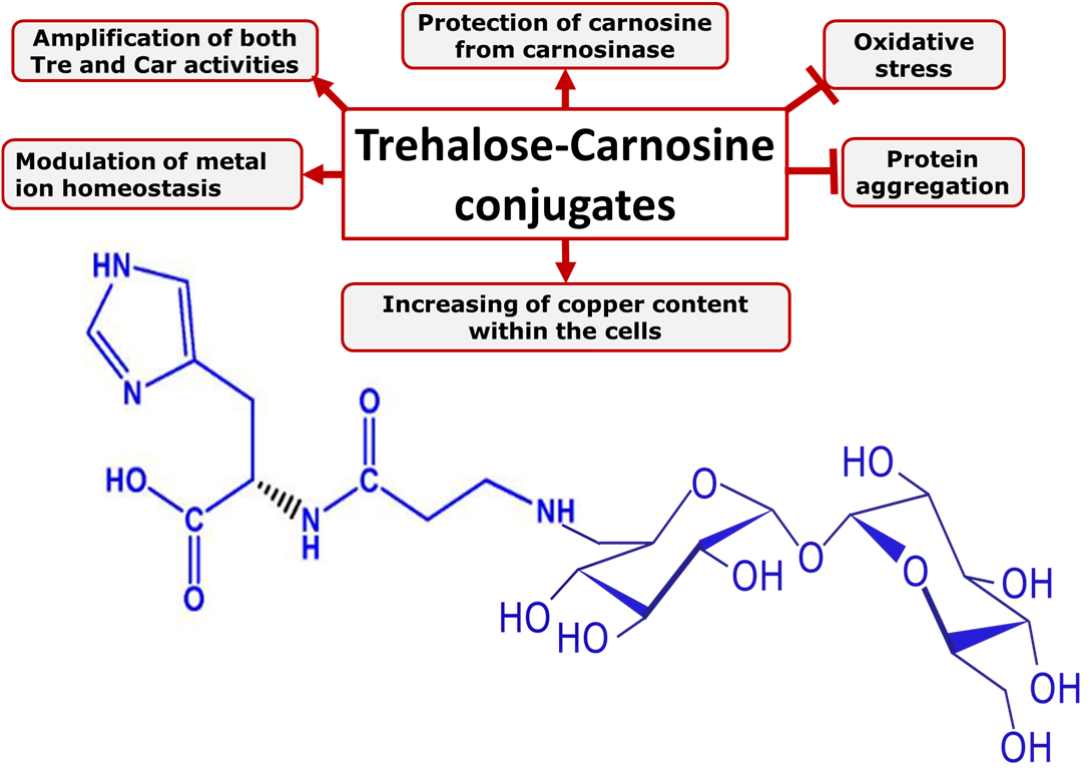
Chemical structure of Tre derivatives.

Glycoconjugates of Car and its analogs with the linear saccharide, Tre, (Figure 6) display several beneficial features in vitro and in vivo [181,182,183,184,315].

In the same context, cyclic polycarbohydrates, cyclodextrins, represent the first family of saccharides functionalized with the dipeptide, equipped with resistance to carnosinase hydrolysis and relevant functions against oxidative stress and protein aggregation, which are the major factors involved in neurodegeneration [183,316,317]. The copper chelating ability and the ROS scavenger protective activities represent an added value for these compounds, as well as the analogs that Vecchio et al. synthesized utilizing other biomolecules [318,319] and mono-(glucose) or di-saccharide (trehalose) as partners in glycoconjugates [320].

**Figure 7:**
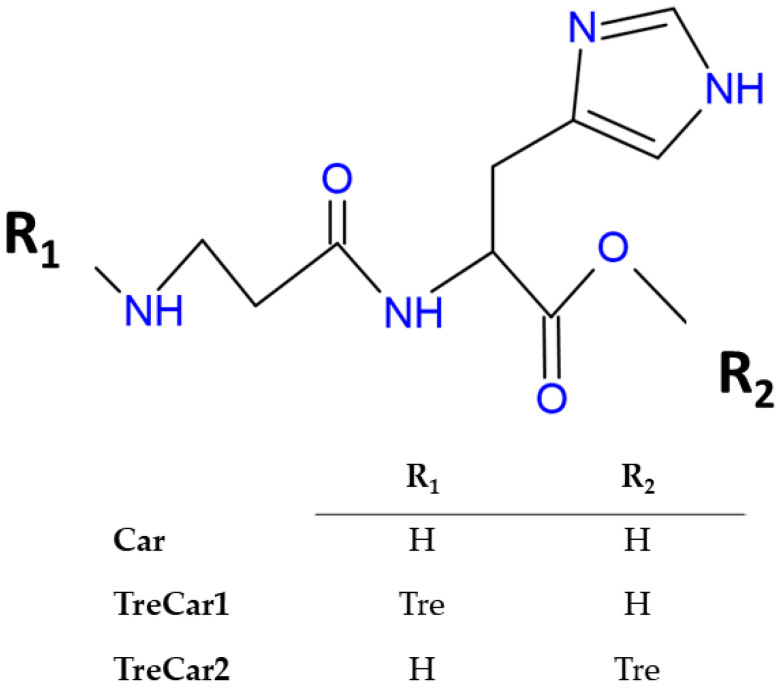
Chemical structure of TreCar1 and TreCar2 (Adapted from Ref. [186]).

Tre is employed to obtain its conjugates with the two diastereomer components of silibinin, silybin A (SilA) and silybin B (SilB), which bind both toxic Aβ (amyloid beta assemblies) and Aβ monomers, inhibiting the formation of amyloid fibers and delaying amyloid polymer growth [321]. A Tre-conjugated peptidomimetic (Ac-LPFFD-Tre) [322] can recognize a hydrophobic domain of Aβ, modify the aggregation features of the polypeptide, and protect neurons from Aβ oligomers’ toxic insult [323]. Different studies report on the glycoconjugates of Tre with carnosine. Initially, the disaccharide was employed to functionalize the amino group of Car, forming TreCar1 [324,325]; then the dipeptide carboxylate group was involved in the conjugation process with Tre, yielding TreCar2 [186] (Figure 7).

**Figure 8:**
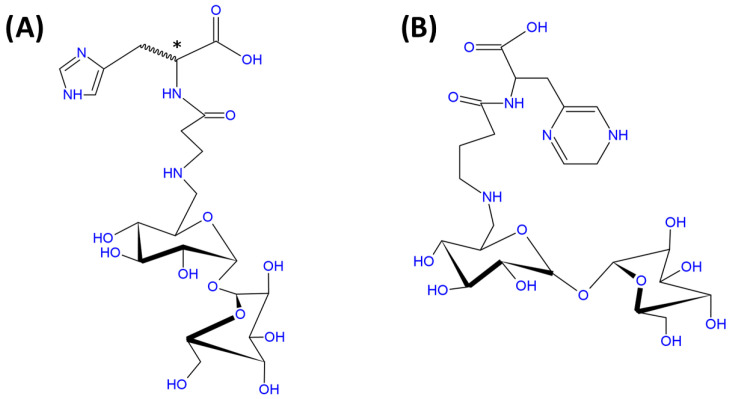
Chemical structure of (**A**) TreLCar (* = L), TreDCar (* = D), and (**B**) TreHCar (Adapted with permission from Refs. [182,326]).

Moreover, D-trehalose-L-carnosine and D-trehalose-D-carnosine were synthesized [325] (Figure 8A) and the glycoconjugation was extended to homocarnosine (TreHCar) [182] (Figure 8B).

This new class of glycoconjugates synergistically combines the preeminent stabilizing, anti-aggregating, and autophagy-inducing activity of Tre with the antioxidant, antiglycating, anti-inflammatory, and metal-chelating functions of carnosine [52]. Furthermore, Tre protects homocarnosine [182] and carnosine [183] from their degradation by carnosinase and potentiates the SOD-like activity of their copper(II) complexes.

The comparative studies of TreCar1 and TreCar2 reveal structural differences that can affect their beneficial effects. [186] The two trehalose derivatives show an analogous protection from carnosinase-induced hydrolysis, demonstrating that both the carboxyl and primary amino groups of carnosine are crucial for carnosinase recognition and, hence, for peptide hydrolysis (Figure 7). On the contrary, the reaction kinetics of the two Tre-carbosine conjugates (TreCar1 and TreCar2) with acrolein (ACR) were characterized by different trends in the ACR amount reduction. Analogously to Car, TreCar2 reduces the ACR percentage to zero within 4 h, while TreCar1 takes approximately twice as long to reduce the ACR content as TreCar2. This difference can be attributed to the structure of the two derivatives, i.e., to the involvement of the primary amino group, which is more efficient at scavenging ACR than other functional groups. Evident differences appear in the Aβ anti-aggregating effect of the two Tre derivatives, with a major efficacy of TreCar1 in comparison to TreCar2, suggesting a significant role of the negatively charged carboxylic group [186]. The influence of negative charges on the inhibition of amyloid aggregation of some compounds is reported in the literature [326,327] and ascribed to the interaction with the ion pairs of Aβ. The copper chelating ability of TreCar1 and TreCar2 is different, but both show similar abilities against metal-driven Aβ fibril formation and interesting SOD1-like activities of the same order of magnitude as other low molecular-weight SOD mimics.

TreCar1 demonstrates antioxidant effect in cell cultures and also in vivo, in a mouse model of spinal cord injury (SCI), where protection of the Car conjugate [315] amplifies the analogous effect of the parent molecules [99]. TreCar1 treatment inhibits inflammatory stress, blocking the inflammatory signaling pathways activated by NF-kB and exerting protective effects on SCI. Anti-apoptotic and trophic activation by TreCar1 was demonstrated by the positive balance between the anti-apoptotic marker Bcl2 and the pro-apoptotic marker Bax [315].

Tre favors the passage of the dipeptide through the cell membrane, increasing the intracellular level of Car by TreCar and improving its function [185]. The disaccharide also potentiates the ionophore function of the dipeptide that binds copper and zinc ions [120], increasing the metal content within the cells [185]. Thus, TreCar1 can transport into the cell copper present at µM levels in the culture medium and in the added serum, as recently demonstrated for analogous systems [147]. Next, the redistribution of copper ions inside the cell activates the protein kinase cascade; phosphorylation of PI3K/Akt and CREB induces the expression of BDNF, VEGF, and GDNF (Glial-Derived Neurotrophic Factor), which are well-known therapeutic options for neurodegeneration [328].

The ionophore ability of TreCar1 [185] affects the expression of different copper and zinc metallostasis network players [141,329]: Ctr1 [139], Sp1 [135], and Zn influx transporters ZnT1 and ZnT3 [330]. In vivo study demonstrates that TreCar1, similar to Car, chelates extracellular zinc ions, influencing ZnT1 and ZnT3 expression [315]; TreCar1 induces metal ion uptake, increasing the cytosolic labile metal ion pool that is involved in signaling pathways.

### 4.2. Glycoconjugates with Hyaluronic Acid

**Figure 9:**
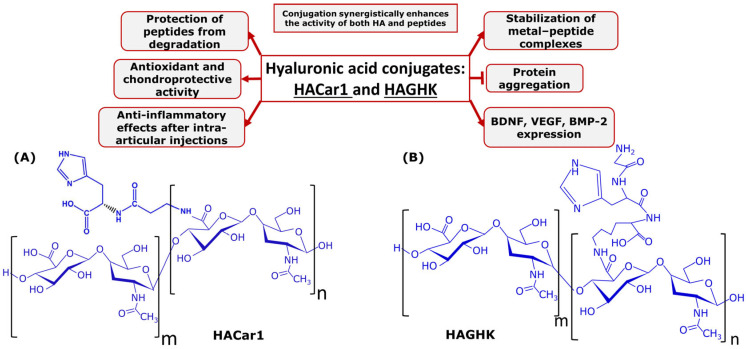
Chemical structure of hyaluronic acid derivatives. (**A**) HACar1 and (**B**) HAGHK.

**Figure 10:**
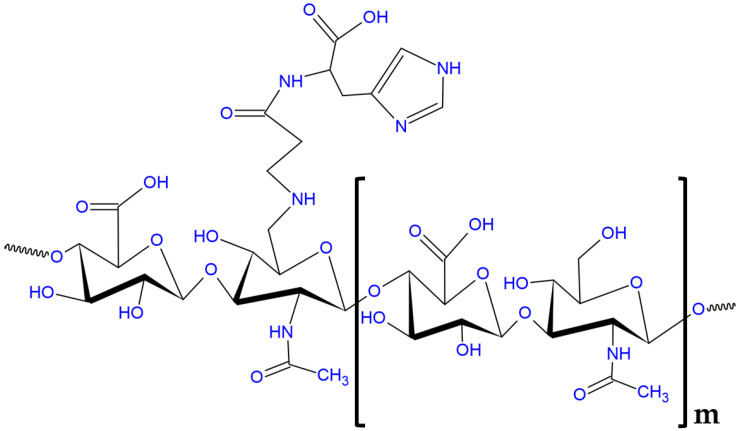
Chemical structure of HACar2, m—number of repeat units. (Adapted from Ref. [335]).

The side chain reactive groups of polysaccharides are advantageous for functionalization with active molecules [330,331,332,333] in an effort to obtain new molecular entities featured by biodegradability, bio-compatibility, and less toxicity. Two different HACar derivatives were synthesized, HACar1 [334] (Figure 9), and HACar2 [335] (Figure 10).

HACar1 synthesis results from a combination of HA at two different molecular weights (200 and 700 kDa) with different loading amounts of Car (Table 1). The glycoconjugates show significant beneficial features due to the synergistic contributions of the two components [113].

Both HACar1(200) and HACar1(700) derivatives treated with carnosinase display a dipeptide molecular integrity that is approximately one order of magnitude greater than that of Car alone or in a mixture with hyaluronic acid [113].

HACar1(200) conjugates inhibit the formation of Ab-amyloid species in a way directly proportional to their concentration, and the higher the Car content covalently linked to the HA(200) scaffold is assayed, the better the inhibition activity. The similar anti-aggregating behavior of HACar1(700)35 derivative is complemented by its ability to dissolve preformed Aβ fibrils; this dissolution capacity is shared by HACar1(200) conjugates. Noteworthy, these activities of HACar1(200) and HACar1(700) are higher than those of free Car or a mixture of HA and Car, suggesting that the conjugation process provides a synergistic effect.

In vitro studies using neuronal cell models indicate that HA-Car1 suppresses both the initiation and elongation phases of Aβ fibril formation. Cytotoxicity assays show that Hy(200) and Hy(700), as well as their Car conjugates, significantly increase cell viability in the presence of Aβ. This effect could be ascribed to a decrease in the toxic oligomer species that directly reduces the Aβ-induced cell toxicity. It is important to note that the percentage of HA saturation with carnosine plays an important role in this process [113].

As HA protects Car from the carnosinase attack, Car inhibits/delays the HA degradation provoked by hyaluronidase in HACar1(200); the inhibition effect of carnosine on the enzymatic process is proportional to the peptide loading of the polymer with HACar1(200)35 being more efficient than HACar1(200)20 [184].

The Car conjugation not only slows down the hyaluronidase-mediated degradation of the HA backbone, but it also affects the dimensions of the final hydrolytic fragments. Indeed, the extensive hydrolysis of HA leads to the formation of biopolymer fragments containing two or three repetitive units, whereas the hydrolytic products of the enzymatic degradation of HACar1(200)20 encompass up to 17 repetitive units.

The antioxidant properties of the HACar1(200) derivatives tested in relation to a stable radical (ABTS) and a nitrogen (NO) reactive species outperform those of the single parent compounds, as well as their non-covalent mixture, indicating that the chemical conjugation induces the synergistic effect of HA and Car. Noteworthy, the enzymatic degradation of HACar1(200)20 induced by hyaluronidase does not significantly affect the antioxidant activity of the conjugates: the hydrolytic products retain their antioxidant properties. HACar1(200)35, and not HA, preserves the function of Car to quench acrolein, a toxic carbonyl species and a product of oxidative stress [184].

HACar1(200) and HACar1(700) derivatives are ligands able to bind Cu^2+^, involving multiple binding sites, which increase with higher dipeptide loading and generate polynuclear copper(II) complexes; Cu^2+^ experiences a 2N_Im_, 2O_COO_ binding mode at physiological pH value [326].

The copper(II) complexes with HACar1(200) and HACar1(700) derivatives show a high O_2_^•−^ scavenging ability. The SOD1-like activity of copper(II) complexes with HA(200) and HA(700) is similar to that displayed by CuCar and comparable to that reported for Cu(HPO_4_) (Table 2) [326].

The I_50_ values of the copper(II) complexes with HACar1(200) and HACar1(700) derivatives indicate that these conjugates possess scavenging capacities comparable to those shown by the native SOD1. The copper(II) complexes with HACar1(200) show an SOD1-like ability, which increases in a Car-loading-dependent manner. This behavior is less evident for the analogous copper(II)complexes with HACar1(700), which also appear less efficient in comparison with the corresponding species of HACar1(200) due to the higher viscosity of the HACar1(700) solutions.

Previous studies [183,325] report the ability of different saccharides, β-cyclodextrin and α,α-trehalose, conjugated with carnosine to affect the O_2_ •− scavenger activity of their related copper(II) complexes, improving the SOD-like activity in comparison with that shown by CuCar. The increased scavenging capacity was ascribed to the interaction of the OH groups, which are present in the saccharides, with the O_2_ •− species, promoting the binding of the superoxide anion radical via the formation of H-bonds, which entrap the O_2_ •− in the right position to interact with the catalytic metal center. These features favor the formation of functional SOD1 mimics with a scavenger effect of the same order of magnitude as that of the native enzyme. Analogously, the same non-covalent interactions between the HA hydroxyl groups and the O_2_ •− radicals can be invoked to explain the high SOD-like capacity of the copper(II) complexes with HACar1 derivatives [326].

HA-Car1(200) derivative interaction with submicromolar copper ions from culture medium stimulates the cellular Nrf2 antioxidant signaling pathway and favors its translocation to the nucleus, giving rise to a protective activity against the oxidative stress induced in specific cells treated with inflammatory agents. Inflammatory conditions depress the Nrf2 signaling pathway by drastically reducing HO-1 expression, but this effect is largely counteracted via treatment with HACar1(200) derivative, and its effect is copper-dependent [326].

Moreover, intra-articular HACar1 injections improve joint lubrication and exert anti-inflammatory effects, partly by downregulating oxidative mediators [336]. HACar1 demonstrates synergistic antioxidant effects vs. single compounds or Car + HA mixture and chondroprotective activity in osteoarthritic rat models. Rats with osteoarthritis, treated with the oral administration of HACar1, show a significant decrease in the levels of pro-inflammatory cytokines and chemokines; in contrast, Car alone, HA, or Car + HA do not provoke a significant effect. HACar1 oral treatment reduces the joint inflammation and cartilage degeneration in vivo in a more significant way compared to that of HA and/or Car [336]. Through these combined effects, HA and its conjugates act as a regulator of oxidative and nitrosative pathways, protecting cells from excessive damage and helping to maintain tissue homeostasis.

To preserve the stability of GHK, different tools have also recently been proposed [46,225,337,338]. To the same aim, a new conjugate HAGHK(200) [146], consisting of GHK and hyaluronic acid (MW 200 kDa), was recently synthesized; the tripeptide and the polysaccharides are linked together through an amide bond between the carboxyl residues spanning the polysaccharide chain and the ε-amino group of the lysine residue of the tripeptide (Figure 9B). The functionalization of the amino group present in the side chain of the lysine allows the conjugated tripeptide to maintain the same potential binding features of GHK.

HAGHK synthesis results from a combination of HA (MW 200 kDa) with different amounts of GHK, corresponding to a loading percentage in GHK of 5, 10, or 15% [146]. The synthesized conjugates show good scavenging activity against ROS, indicating that conjugation stabilizes the antioxidant activity of the tripeptide alone. Moreover, the copper(II) complexes with HA or GHK show SOD1-like activity; in analogy to what was reported above for the metal complexes with HACar1(200) or HACar1(700) derivatives, the copper(II) complexes with HAGHK(200) derivatives display a higher SOD1-like activity in comparison with those found for the copper(II) complexes with HA or GHK. This finding highlights that the conjugation potentiates the superoxide radical anion (O_2_ •−) scavenging ability of GHK-Cu(II). This interesting SOD1-mimicking activity of the new conjugates can be explained by bringing into play the HA hydroxyl groups, which can form a network of hydrogen bonds with the superoxide radical anions, thus favoring their interaction with the catalytic copper ion. It is reasonable to assume that the OH groups mimic the role of positively charged amino acids in the native SOD1 through a process known as “electrostatic tunneling”. As shown in the case of all HACar1 conjugates, the efficiency of SOD1 mimetics is also related to the flexibility of the ligand. This allows the donor atoms to arrange around the metal center during the catalytic process, which is featured by a change in oxidation state of copper, from Cu^2+^ to Cu^+^, metal ions that require different coordination geometries.

The HAGHK(200) conjugates show not only chemical but also biological protective effects higher than those of the mixture of its components, highlighting the synergistic role of the covalent coupling of GHK with HA. In in vitro assays, the cellular treatment with HAGHK(200) derivatives put in evidence that the conjugates can bind copper (II) ions present in the culture medium; the metal high affinity of HAGHK(200) derivatives induces an increase in the activity of both HA and GHK, in terms of expression and release of trophic proteins [146].

Until 2008, it was considered that copper transport could only occur in the cytoplasm and membranes, and there was no specific copper chaperone that transported Cu^+^ ions into the nucleus. However, the work of Itoh et al. [339] changed this paradigm by showing that Atox-1 functions not only as a classical cytosolic Cu chaperone, delivering copper to ATP7A/B when intracellular copper content increases, but also as a copper-dependent transcription factor capable of binding to gene promoter regions and regulating cell proliferation and stress response.

Copper transport may take place in several organelles, as evidenced by the presence of CCS and SOD1 in the nucleus, mitochondria, lysosomes, and peroxisomes [340,341,342]. CCS accumulation in the nucleus [341] occurs under hypoxic conditions, is copper-dependent, and contributes to the hypoxia-inducible factor-1α activation [343,344], which in turn affects VEGF and lysyl oxidase expression [345]. Copper forms a complex with ionophore peptides [147], influences the decrease in the CCS in the cytoplasm, while significantly increasing its nuclear concentration even under normoxic conditions. This indicates a mechanism of interaction between copper and the peptide that promotes the penetration of CCS into the nucleus beyond the pathways caused by hypoxia. In the nucleus, CCS acts as a chaperone, and as a recent study shows [191], functions as a transcription factor, which corresponds to the dual behavior of a chaperone/transcription factor, previously also established for Atox-1 [339].

The ionophore role of HAGHK(200) favors the copper uptake by cells, stimulating intracellular metal signaling pathways of the kinase cascade, according to recent findings [147]. Thus, using endothelial and fibroblast cell models [146], it was shown that copper-driven HAGHK(200) induces the expression or/and release of VEGF, the major angiogenic factor, BDNF, whose are well known physiological functions and therapeutic potential in depression, neurodegeneration and brain cancer [346], and the BMP-2, a key osteogenic factor [347], also involved in the formation of appropriate synaptic connections and development of normal neural circuits in the brain [348]. Moreover, the main copper homeostasis players [141], the high-affinity cellular membrane copper transporter 1, Ctr1 [139], and intracellular copper chaperones such as CCS and Atox-1 [143], play a key role in these processes. Ctr1, which modulates the majority of copper influx in the cell, is required for different trophic factor signals; CCS and Atox1 translocate to the nucleus and act as transcription factors regulating the expression of above mentioned trophic and angiogenic genes.

## 5. Conclusions

The synthesis of carnosine glycoconjugates with different carbohydrates (cyclodextrins, glucose, sucrose, trehalose, and hyaluronan) produces inhibitors of human serum carnosinase, potentiating the endogenous dipeptide protective activities. Among these, particularly noteworthy are the recent findings which highlight for the first time that endogenous Car plays an antioxidant role in vivo and protects oligodendrocytes against oxidative stress, attenuating myelin loss [349]. Furthermore, recent transcriptome studies indicate a perturbed, mostly decreased, carnosine synthetase expression in demyelinating disorders, including multiple sclerosis [350,351,352]. Within the CNS, however, the absence of Car leads to larger lesions with massive neuroinflammation, demyelination, and neuronal axonal damage, which are partially rescued by exogenous carnosine administration [353]. Analogously, the stabilization of GHK-Cu by glycoconjugation recalls a recent report on the use of new biotinylated GHK and related copper(II) complex for neurodegenerative disorders [230], offering new potentiated stimuli to the neuroprotective activities of GHK/GHK-Cu [354,355,356,357,358].

In some cases, the protective activities carried out in vitro refer to GHK, but it is reasonable to accept the suggestion that the active species is GHK-Cu formed by GHK interaction with the metal ion present in the culture medium and in the added serum [205]. The occurrence of an analogous involvement of copper is generally overlooked in in vitro findings, as well as in the rationalization of the protective abilities of Car application for the treatment of several diseases, including neurodegeneration [71,117,359,360,361]. The appreciation of metal binding and ionophore ability of small peptides, together with the capacity of elements of d-block elements to activate relevant signaling pathways [180], allowed for a more complete description of the neuroprotective functions of NT mimics [145,147,164,165,166,167,168,169,362]. Analogously, the two endogenous peptides, Car and GHK, and their glycoconjugates favor cellular uptake of added or culture medium copper that translates at the nucleus as CCS and Atox1, where the two chaperones support or act as transcription factors originating the expression/release of neurotrophic and angiogenic proteins. Furthermore, the contribution of the disaccharide Tre and polysaccharide HA is more extensive than initially planned in view of obtaining efficient delivery systems; both saccharides share with the endogenous peptides their antioxidant, anti-inflammatory, and anti-aggregating features and their ability to affect the bioavailability of metal ions [269,363]. In addition, different studies report on the HA [364,365] and Tre [366,367,368] abilities to promote neuroprotection in different brain diseases, extending the sharing with the peptides of the above highlighted functions, and paving the way to potential developments of these glycoconjugates for treatments against neurodegenerative disorders.

## Figures and Tables

**Table 1:** List of HACar1 derivatives. The average number of repeat units conjugated or not to Car (*n* and *m*, respectively) was calculated on the basis of the structural characterization ^(a)^.

	Car Loading			
	HA MW	%	*m*	*n*
HA(200)	200	-	510	-
HACar1(200)7	200	7	475	35
HACar1(200)10	200	10	459	51
HACar1(200)14	200	14	440	70
HACar1(200)35	200	35	335	175
HA(700)	700	-	1965	-
HACar1(700)35	700	35	1285	680

^(a)^ Adapted from Ref. [113].

**Table 2:** SOD1-like activity of: (i) the copper(II) complexes with HA(200), HA(700), and Car; (ii) the copper(II) complexes with HACar1 derivatives with different MW and Car loading; and (iii) SOD1 and copper(II) complexes with phosphate.

SOD1/SOD1 Mimic	I_50_ µM
SOD1	0.014(3)
Cu-HACar1(700)10	0.05(4)
Cu-HACar1(700)25	0.05(2)
Cu-HACar1(700)35	0.09(2)
Cu-HACar1(200)7	0.21(4)
Cu-HACar1(200)10	0.09(1)
Cu-HACar1(200)14	0.03(2)
Cu-HACar1(200)25	0.03(2)
Cu-HACar1(200)35	0.02(1)
Cu-HA(700)	0.8(2)
Cu-HA(200)	1.0(3)
CuCar	1.0(2)
Cu(HPO_4_)	1.06

I_50_ (the concentration that causes the 50% inhibition of the reduction in nitro blue tetrazolium chloride as a detector molecule of O_2_) of the copper (II) complexes at pH 7.4 (Adapted from Ref. [326]).

## Data Availability

No new data were created or analyzed in this study. Data sharing is not applicable to this article.
